# Hyposmia as a Predictive Marker of Parkinson's Disease: A Systematic Review and Meta-Analysis

**DOI:** 10.1155/2019/3753786

**Published:** 2019-05-19

**Authors:** Xin Sui, Changli Zhou, Jinwei Li, Lei Chen, Xige Yang, Feng Li

**Affiliations:** ^1^School of Nursing, Jilin University, Changchun, Jilin 130021, China; ^2^The First Hospital of Jilin University, Changchun, Jilin 130021, China

## Abstract

**Background:**

Hyposmia is one of the most common and best-characterized conditions that is also one of the first nonmotor features of Parkinson's disease (PD). The association of hyposmia with PD is widely accepted; however the likelihood of developing PD is unclear. Our meta-analysis aimed to investigate the risk of PD in individuals with hyposmia.

**Methods:**

Prospective studies on humans published before December 4^th^, 2018, were searched for in PubMed, Embase, Web of Science, and Cochrane Library databases. Two independent reviewers screened studies for inclusion and extracted data. We assessed the quality of studies using the Newcastle–Ottawa Scale and pooled data for analysis using random-effects models.

**Results:**

Of the 1774 studies retrieved, seven met the inclusion criteria for this review. A total of 3272 hyposmia and 176 PD events were reported over follow-up periods ranging from 3 to 17 years. Hyposmia was associated with a 3.84-fold risk of developing PD (pooled relative risk: 3.84, 95% CI 2.12−6.95). Subgroup analyses identified few differences between different hyposmia assessment methodologies and follow-up periods.

**Conclusions:**

Our findings suggest that deficiencies in olfaction are associated with an increased risk of developing PD. Future studies are needed to investigate whether hyposmia is a promising and feasible biomarker for the early diagnosis of PD.

## 1. Introduction

Parkinson's disease (PD) has affected an estimated 10 million individuals worldwide and is the second most prevalent neurodegenerative disease. The number of PD patients is estimated to double by the year 2030 owing to the aging population [1–3], which is becoming a great national burden in many countries [4–6]. It has been reported that the incidence of PD has grown to at least 1.5 million in the United States [7] and more than 1.7 million in China [7, 8].

PD is characterized by motor symptoms such as tremor at rest, bradykinesia, rigidity, and postural instability [9, 10]. In addition to motor dysfunction, patients also suffer a variety of nonmotor symptoms, including rapid eye movement sleep behavior disorder [11, 12], hyposmia [13], constipation [9, 10, 14], depression [15, 16], anxiety [15, 17], and excessive daytime sleepiness [18, 19]. Research has demonstrated that when PD is diagnosed clinically after developing motor dysfunction (e.g., nonmotor symptoms), an extensive loss of dopaminergic neurons in the substantia nigra has already occurred [20]. Studies have also indicated that, in more than 90% of PD patients, nonmotor symptoms (such as hyposmia) precede the motor symptoms [21] and even precede the diagnosis of PD by years [22]. Therefore, nonmotor symptoms may be potential early diagnostic indicators and so we postulated whether PD could be predicted earlier by factors other than motor symptoms. Hyposmia exhibits a high prevalence and occurs early in the development of PD. Thus we investigated whether an olfactory test could be a promising tool for predicting PD, as this would enable early diagnosis and thus slow down the progression of PD.

Numerous studies have already indicated a connection between unexplained hyposmia and the later development of PD [13, 23–27], which suggests that a segment of the population with olfactory dysfunction is at a higher risk of suffering PD. Haehner et al. [25] supported the idea of olfactory dysfunction as a very early sign of idiopathic Parkinson's disease. Xiao et al. [27] stated that, in addition to its high prevalence, the occurrence of hyposmia may also predict a higher risk of PD. Recently, more and more studies are investigating hyposmia as a potential screening tool that may play an important role in detecting the risk of developing PD.

However, the process of using olfactory tests to predict PD can be challenging. Xiao et al. [27] concluded that olfactory tests can only be used to differentiate idiopathic PD from other diseases. Other studies have shown that patients exhibit normal or nearly normal olfactory function when they develop progressive supranuclear palsy [28, 29], MPTP-induced parkinsonism [28], multiple system atrophy [29], corticobasal degeneration [29], and essential tremor [30], which suggests that olfactory testing cannot discriminate between these diseases and PD. Therefore, although several studies have suggested that olfactory testing is a valuable tool for diagnosing PD, we still need more high-quality studies to further confirm the accuracy and reliability of olfactory disorders for Parkinson's predictions.

To date, no meta-analyses have examined the magnitude of the association between the development of hyposmia and the risk of PD. We therefore conducted a meta-analysis of this issue on prospective studies to provide more information on whether hyposmia can be a potential biomarker and predictor for the early detection and diagnosis of PD.

## 2. Materials and Methods

### 2.1. Search Strategy

A systematic and comprehensive literature search of articles that investigated the association between hyposmia and PD was conducted by two independent researchers (XS and FL) using the electronic databases PubMed, Web of Science, Embase®, and Cochrane Library. All articles collected during the search were published in English from the start date of the database to December 4^th^, 2018. Keywords on PD and hyposmia were searched for using the medical subject heading (MeSH) terms “Parkinson Disease” and “Olfaction Disorders”, the PD-related terms “Idiopathic Parkinson's Disease”, “Lewy Body Parkinson Disease”, “Lewy Body Parkinson's Disease”, “Primary Parkinsonism”, “Parkinsonism, Primary”, “Parkinson Disease, Idiopathic”, “Parkinson's Disease”, “Parkinson's Disease, Idiopathic”, “Parkinson's Disease, Lewy Body”, “Idiopathic Parkinson Disease”, and “Paralysis Agitans”, and the olfactory-related terms “Olfaction Disorder”, “Smell Disorders”, “Smell Disorder”, “Cacosmia”, “Cacosmias”, “Dysosmia”, “Dysosmias”, “Paraosmia”, “Paraosmias”, “Anosmia”, “Hyposmia”, “Olfactory Hallucination”, and “Smelling Disorder”. After removing duplicates, the titles and abstracts of potentially relevant articles were screened and assessed for their suitability by two researchers (XS and FL). Articles with abstracts that did not mention hyposmia and PD were excluded. The entire length of each article considered suitable was obtained and reviewed to determine its eligibility for inclusion or exclusion. Their results were compared, and disagreements were resolved by a discussion and consensus. In addition, the references in the reviews that were retrieved in the original search were examined for more articles, which were then subjected to the same filtering process as that described above.

### 2.2. Inclusion Criteria

Articles were considered eligible if they met the following criteria: (1) reported prospective studies involving humans, (2) examined patients with PD who had undergone a hyposmia test before their PD diagnosis, and (3) reported the relative risks (RRs), odds ratios (ORs), and/or hazard ratios (HRs) with 95% confidence intervals (CIs) or reported original data that could be used to calculate RR, OR, and HR with 95% CIs (CI). We did not limit the included studies with regard to their methodology for diagnosing hyposmia and PD, so as to ensure a large sample size.

### 2.3. Exclusion Criteria

Articles were excluded if they met any of the following criteria: (1) published as a case-control, cross-section design, case report, review, conference abstract, comment, or letter, (2) reported only risk estimates (RR, OR, or HR) without reporting 95% CIs, (3) did not report sufficient data to calculate the risk estimates, (4) consisted of duplicate populations, (5) were non-English publications, or (6) were not published.

### 2.4. Data Extraction and Quality Assessment

The data of each study were extracted into a predesigned form that included the following information: name of first author, year of publication, country, population, study data, sample size, PD diagnosis, hyposmia assessment method, and follow-up period. Finally, the quality of the studies was evaluated using the Newcastle–Ottawa Scale (NOS) [30], which rates eight aspects of each study for a maximum score of nine points.

### 2.5. Statistical Analysis

The effect measures were combined using standard meta-analysis methods. We used a random-effects model to calculate the RR, OR, or HR as the metric of risk and 95% CI. The RR, OR, or HR from each study was weighted using the Mantel-Haenszel procedure.

The statistical heterogeneity across the studies was evaluated by I^2^ tests, which assess the appropriateness of the pooling of the results of the individual studies. The I^2^ test estimates the amount of variance across the studies resulting from heterogeneity rather than chance. The heterogeneity was considered substantial if I^2^ > 50%. Moreover, we performed subgroup analyses to investigate heterogeneity across the studies. The studies were divided into groups according to their hyposmia assessment.

Sensitivity analyses were performed to test the source of heterogeneity. Potential publication bias was evaluated using Begg's and Egger's tests [31], and p < 0.05 was considered to indicate publication bias. The statistical analyses were performed using Stata (version 12.0; StataCorp LLC, College Station, TX, USA) and RevMan (version 5.3; The Cochrane Collaboration, London, UK).

## 3. Results

### 3.1. Literature Search

**Figure 1:**
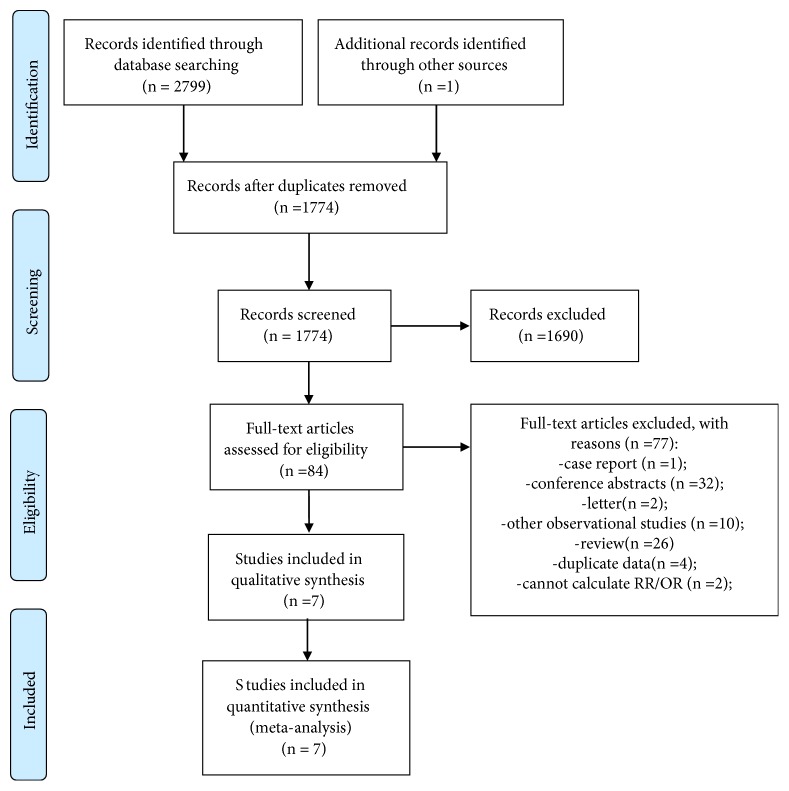
Flow chart for identifying eligible studies.

After the removal of duplicates, the literature search yielded 1774 articles, including one article found in a reference list. Of these, 1690 articles were excluded after reviewing their title and/or abstract. After reviewing the remaining 84 full-length articles, 77 articles were excluded for the following reasons: formatted as a case report (n=1), conference abstract (n=32), letter (n=2), other observational studies that were not prospective studies (n=10), or review (n=26), and reported inadequate data for calculating RR/OR (n=2), or contained duplicate datasets (n=4). A final total of seven studies that fulfilled our inclusion criteria were included in our meta-analysis (Figure 1). The main characteristics of the articles analyzed are summarized in Table 1.

### 3.2. Sample Characteristics

The seven articles contained potential factors that could have influenced the results including age, gender, and country. The age ranged from 26 to 95 years old. Four studies [32–34, 36] examined more females than males, while another study [35] examined more males. One study [13] examined only men and one study [37] did not clarify the gender distribution. The studies were performed in the following countries: Germany [32, 35], Italy [35], Austria [35], US [13, 34], UK [33], Australia [36], the Netherlands [37], and Japan [13].

#### 3.2.1. Assessment of Hyposmia

In the seven papers on hyposmia, five different tools were used: the “Sniffin' Sticks” [32, 35], the Brief Smell Identification Test (BSIT) [13, 34], the US version of the University of Pennsylvania Smell Identification Test (UPSIT) [33], the San Diego Odor Identification Test (SDOIT) [36], and a combination of an odor detection, discrimination, and identification task [37].

#### 3.2.2. Ascertainment of PD

A total of 176 PD events were recorded across the seven papers. Different methods were used to diagnose PD, including a version of the Unified Parkinson's Disease Rating Scale [13, 35], UK Brain Bank diagnostic criteria [33, 37], and ICD-9-CM [34]. The assessment of PD was unclear in two studies [32, 36]. All studies scored at least 8/9 on the Newcastle–Ottawa Scale of quality.

#### 3.2.3. Hyposmia and the Risk of a PD Outcome

**Figure 2:**
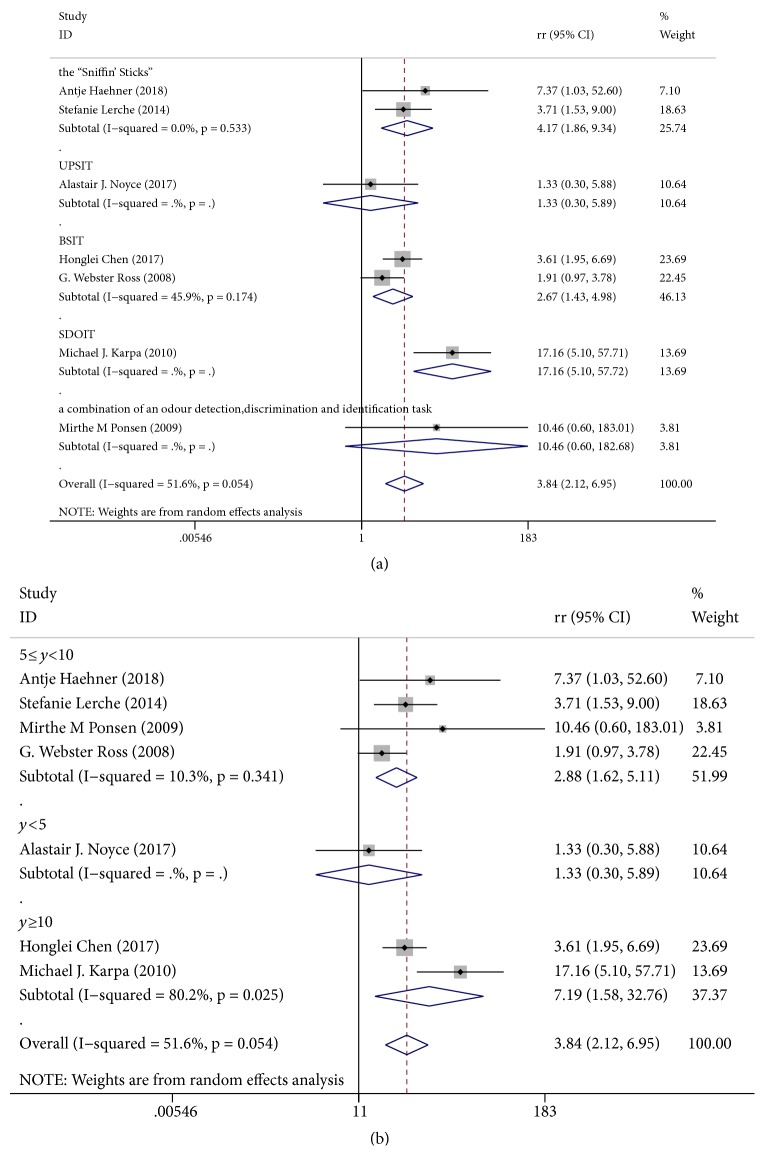
Forest plots of studies investigating the incidence of PD. (a) Subgroup analysis according to different hyposmia assessments. (b) Subgroup analysis according to different follow-up periods. UPSIT: the US version of the University of Pennsylvania Smell Identification Test; BSIT: the 12-item Brief Smell Identification Test; SDOIT: the San Diego Odor Identification Test.

For prospective cohort studies, the RR value is most appropriate. Across seven independent study samples (176 events), the average RR of new PD when comparing hyposmia versus normosmia was 3.84 (95% CI 2.12−6.95; Figures 2(a) and 2(b)). The outcome showed that, during the follow-up period, hyposmia was significantly associated with an increased risk of PD. Although little evidence of heterogeneity was found within this comparison (I^2^=51.6%, p=0.054), we still explored whether this could be explained by different hyposmia assessments and different follow-up periods. We found evidence that effects differed according to each subgroup. However, we were not able to explore whether different ages and genders were potential sources of heterogeneity due to insufficient data.

#### 3.2.4. Publication Bias

**Figure 3:**
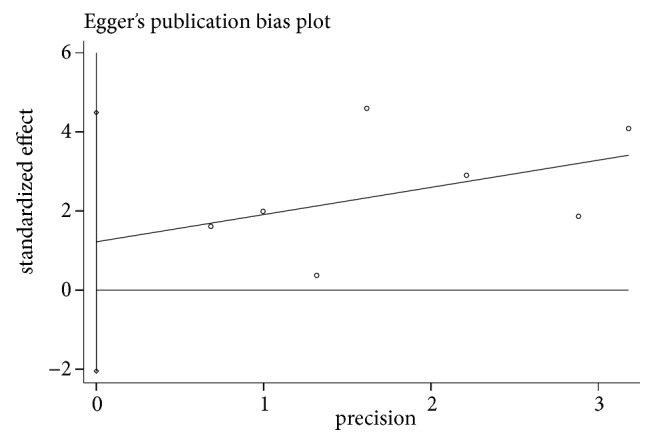
Egger's publication bias plot.

Begg's and Egger's tests were used to test for publication bias and the outcome exhibited no publication bias (Begg's Test: p = 0.548; Egger's test: p = 0.381; Figure 3).

#### 3.2.5. Sensitivity Analysis

**Figure 4:**
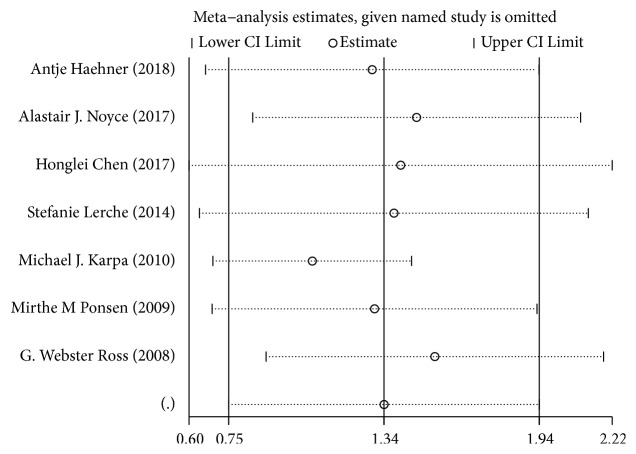
Sensitivity analysis for the included studies.

To identify the studies that may have contributed to the pooled heterogeneity, we removed each of the seven studies, one at a time, and detected the magnitude of the pooled RR after the removal of each study. Such analyses revealed that the association between hyposmia and the risk of developing PD was still statistically significant (Figure 4).

The results of our meta-analysis demonstrated that PD and hyposmia exhibited a definite association. Furthermore, hyposmia that occurred before the PD diagnoses exhibited a 3.84-fold higher risk of developing PD.

## 4. Discussion

PD has become a recognized disease worldwide and is characterized by motor and nonmotor symptoms. PD is always diagnosed after the occurrence of the cardinal motor signs during clinical practice, even though most of the nonmotor symptoms tend to appear before the motor symptoms of PD. Thus we postulated whether nonmotor symptoms could be utilized as a tool for the early diagnosis and even possible prevention of PD. Hyposmia is one of the most common and best-characterized nonmotor features and is often one of the first manifestations of PD [7, 23, 38, 39]. Therefore, the purpose of this paper was to explore whether hyposmia could be considered an early predictive indicator of PD.

Our systematic review and meta-analysis were performed to investigate the association between hyposmia and PD. The meta-analytic pooling under the random-effects model showed that hyposmia was significantly associated with an increased risk of the development of PD during the follow-up period. Compared with healthy controls, individuals with symptoms of hyposmia had a 2.12−6.95-fold increased risk of developing PD. In addition, the heterogeneity of the seven studies was relatively low and the results were steady and credible.

Multiple lines of evidence have suggested that hyposmia is a predictive indicator of PD. Ponsen et al. [23] indicated that idiopathic olfactory dysfunction was associated with an increased risk of developing PD by at least 10%. Ross et al. [13] also found that hyposmia can predate clinical PD by at least four years among men. Data from Haehner et al. [25] suggested that a combination of olfactory testing and other tests may constitute a screening tool for the risk of developing PD, which is consistent with our findings. However, different time periods until follow-up may affect such results. Accordingly, we performed a subgroup analysis of seven articles with different hyposmia assessment methods and different follow-up periods. Only a few studies used UPSIT, SDOIT, or a combination of an odor detection, discrimination, and identification task (Figure 2(a)), so the results of these subgroups were not statistically significant. Once assessed by the “Sniffin' Sticks” or BSIT, the I^2^ value became smaller. Thus there is reason to believe that a standardized hyposmia evaluation index would improve the predictive value of hyposmia in diagnosing PD. Subgroup analysis in accordance with the follow-up period demonstrated that studies with follow-ups from 5−10 years exhibit lower heterogeneity, whereas studies with follow-ups after > 10 years exhibit much higher heterogeneity. This is expected, as it is inevitable that any heterogeneity increases with time.

The results of our study demonstrated no potential publication bias based on the outcomes of the statistical tests (Begg's Test, p = 0.548; Egger's test, p = 0.381), demonstrating the robustness of the results. In the sensitivity analysis, we removed each study in turn to determine whether it had a significant impact on the overall outcome. However, the results were still in the pooled confidence interval, indicating that no articles constituted a significant bias towards the overall results. All the seven included studies demonstrated good quality, scoring at least 8/9 according to the Newcastle–Ottawa Scale. The risk estimates calculated in this meta-analysis were also fairly strict estimates to further enhance the robustness and credibility of the results of this study.

Olfactory function might serve as a useful indicator to improve the diagnostic processes underlying the early detection of PD, as the results of this analysis on hyposmia suggest. Thus, paying attention to the symptoms of hyposmia will benefit early detection and screening of PD.

### 4.1. Strengths and Limitations

The studies included were found by using a broad, comprehensive, and systematic search strategy of the relevant information. The meta-analysis included seven high-quality prospective studies. Many participants and long follow-ups were analyzed, which minimized potential sampling error and provided enough power to detect the association between early diagnoses of PD and hyposmia. The present results demonstrate that hyposmia is a risk factor for the development of PD and thus expand our understanding of hyposmia and PD. The strict and comprehensive inclusion and exclusion criteria ensured that the included study populations were as homogeneous as possible. In addition, a precise statistical analysis was performed to clarify the significance of the quantitative estimate of the association. We conducted a subgroup analysis based on different hyposmia assessments and different follow-up periods, which helped to clarify the consistency and robustness of the results.

However, our study has several limitations. First, we excluded the gray databases and limited our search to only English publications, which may have resulted in a loss of potentially relevant studies. The analyses of this study estimated that the results were reliable and robust with medium heterogeneity and that there was no publication bias among the studies. However, the variable age, gender, population, methodological assessments of hyposmia and PD, and follow-up periods were unavoidably all confounding factors that potentially affected the outcome. Although subgroup analyses were performed on different hyposmia assessment methods and follow-up periods, it was not performed according to age, gender, population, and different diagnostic criteria of PD because of insufficient data. Apart from the above, most of the articles we searched had positive outcome, some negative results were not reported inevitably. Hence, more articles that publish objective results in the future are needed.

### 4.2. Implications

Olfactory testing is a reliable diagnostic tool that is inexpensive, noninvasive, and quickly and easily performed [25]. Our results suggest that olfactory function might serve as a biomarker of individuals who are at risk of developing PD or who are in the premotor phases of the disease [4, 20]. The magnitude of the association between hyposmia and subsequent PD should be quantified in the future, as this association might help efforts to identify high-risk individuals.

However, due to the limited number of studies in this analysis, we were not able to conclude that hyposmia is a solid predictor of PD. Although this biomarker is promising, the prediction of PD by hyposmia has not yet been unequivocally demonstrated [20, 40]. Even though hyposmia as a biomarker that exhibits high sensitivity, it also exhibits relatively low specificity, as it can be present in individuals with neurological diseases other than PD [27], which limits its diagnostic application. Hence, further prospective studies are needed to examine the presence of hyposmia and its predictive value of PD in order to better understand the etiology of PD.

## Figures and Tables

**Table 1:** Characteristics of the included evidence.

Author	Year	Country	Population	Age	Gender (M/F)	Tevent	Tsample	Cevent	Csample	PD diagnosis	Hyposmia assessment	Follow-up	NOS
Antje Haehner [32]	2018	Germany	patients diagnosed with an idiopathic smell disorder at Smell and Taste Clinic from 2000 to 2014 were contacted for a telephone interview.	70.1 ± 11.4 years (range 26–95 years)	216/258	44	406	1	68	NA	the “Sniffin' Sticks”	average 8.1 years (range 2–17 years; ± SD 3.75)	9
Alastair J. Noyce [33]	2017	UK	participants were recruited via the study website following an advertising campaign in 2011	66.2 (63.5-70.5) years	517/806	4	198	3	198	U.K. Brain Bank diagnostic criteria	UPSIT	3 years	9
Honglei Chen [34]	2017	US	3,075 well-functioning community-dwelling individuals in the metropolitan areas of Pittsburgh, Pennsylvania, and Memphis, Tennessee.	70–79 years	48.4/51.6 (%)	26	764	16	1698	ICD-9-CM	BSIT	16 years	8

Stefanie Lerche [35]	2014	Germany & Italy & Austria	the PRIPS cohorts & Bruneck cohort	59 (50–93) years	51.9/48.1 (%)	12	359	8	888	the Unified Parkinson's Disease Rating Scale (UPDRS-I)	the “Sniffin' Sticks”	5 years	9
Michael J. Karpa [36]	2010	Australia	the BMES cohort	>60 years	685/951	19	441	3	1195	NA	SDOIT	10 years	8
Mirthe M Ponsen [37]	2009	The Nether-lands	cohort of non-parkinsonian, non-demented first-degree relatives of PD patients	NA	NA	5	40	0	38	the UK Parkinson's disease Society Brain Bank (UK-PDSBB) criteria	a combination of an odour detection, discrimination and identification task	5 years	9
G. Webster Ross [13]	2008	US & Japan	Honolulu-Asia Aging Study (HAAS)	79.7 ±4.1 (range, 71–95) years	men	22	1064	13	1203	the Unified Parkinson's Disease Rating Scale	BSIT	8 years	8

M/F: male/female; Tevent: people with PD and hyposmia; Tsample: people with PD; Cevent: people with PD but without hyposmia: Csample: people without PD or hyposmia; NOS: the Newcastle–Ottawa Scale; NA: not clear; UPSIT: the US version of the University of Pennsylvania Smell Identification Test; ICD-9-CM: International Classification of Diseases, 9th Revision, Clinical Modification; BSIT: the 12-Item Brief Smell Identification Test; SDOIT: the San Diego Odor Identification Test.
